# Colonization with multidrug resistant organisms determines the clinical course of patients with acute myeloid leukemia undergoing intensive induction chemotherapy

**DOI:** 10.1371/journal.pone.0210991

**Published:** 2019-01-23

**Authors:** Olivier Ballo, Ikram Tarazzit, Jan Stratmann, Claudia Reinheimer, Michael Hogardt, Thomas A. Wichelhaus, Volkhard Kempf, Hubert Serve, Fabian Finkelmeier, Christian Brandts

**Affiliations:** 1 Department of Medicine, Hematology/Oncology, Goethe University Hospital, Frankfurt/Main, Germany; 2 University Cancer Center Frankfurt (UCT), Goethe University Hospital, Frankfurt/Main, Germany; 3 Institute of Medical Microbiology and Infection Control, Goethe University Hospital, Frankfurt/Main, Germany; 4 University Center for Infectious Diseases, Goethe University Hospital, Frankfurt/Main, Germany; 5 Department of Medicine, Gastroenterology, Hepatology and Endocrinology, Goethe University Frankfurt, Frankfurt/Main, Germany; University of Kentucky, UNITED STATES

## Abstract

**Introduction:**

The global spread of multidrug-resistant organisms (MDRO) complicates treatment and isolation measures in hospitals and has shown to increase mortality. Patients with disease- or therapy-related immunodeficiency are especially at risk for fatal infections caused by MDRO. The impact of MDRO colonization on the clinical course of AML patients undergoing intensive induction chemotherapy—a potentially curative but highly toxic treatment option—has not been systematically studied.

**Materials & methods:**

312 AML patients undergoing intensive induction chemotherapy between 2007 and 2015 were examined for MDRO colonization. Patients with evidence for MDRO before or during the hospital stay of induction chemotherapy were defined as colonized, patients who never had a positive swab for MDRO were defined as noncolonized.

**Results:**

Of 312 AML patients 90 were colonized and 130 were noncolonized. Colonized patients suffered from significantly more days with fever, spent more days on the intensive care unit and had a higher median C-reactive protein value during the hospital stay. These findings did not result in a prolonged length of hospital stay or an increased mortality rate for colonized patients. However, in a subgroup analysis, patients colonized with carbapenem-resistant enterobacteriaceae (CRE) had a significantly reduced 60- and 90-day, as well as 1- and 2-year survival rates when compared to noncolonized patients.

**Conclusion:**

Our analysis highlights the importance of intensive MDRO screening especially in patients with febrile neutropenia since persisting fever can be a sign of MDRO-colonization. CRE-colonized patients require special surveillance, since they seem to be at risk for death.

## Introduction

Acute myeloid leukemia (AML) is a hematological malignancy of the myeloid blood lineage. Due to the fatal course of this aggressive disease a curative therapy approach can only be achieved by intensive induction chemotherapy, usually consisting of cytarabine in combination with an anthracycline [1]. Due to its high toxicity this treatment protocol is reserved for younger patients with limited comorbidities [2]. Standard induction chemotherapy for these patients—excluding the unique treatment for acute promyelocytic leukemia subtype—contains cytarabine combined with an anthracycline such as daunorubicin [3]. Treatment related mortality (TRM) is seen in about 4.5% of these patients [4]. Bacterial infections are the most common cause for TRM in these patients and in patients with chemotherapy-induced neutropenia in general [5].

The global spread of multidrug-resistant organisms (MDRO), namely vancomycin-resistant enterococcus (VRE), methicillin-resistant staphylococcus aureus (MRSA) and multidrug-resistant gram-negative bacteria (MDRGN) complicates treatment and isolation measures in hospitals. In a study by Sostarich *et al*. from the University Hospital Aachen an increased mortality rate and prolonged stay on intensive care unit (ICU) in patients with bloodstream infections (BSI) by MDRO was observed [6].

The immunodeficiency associated with leukemia as well as with intensive chemotherapy leads to prolonged episodes of neutropenia, which in turn may further increase TRM for AML patients if colonized with a MDRO. The impact of MDRO-colonization on patients undergoing intensive induction chemotherapy for AML has not been systematically studied. We hypothesized that colonization with a MDRO affects the clinical course of AML patients undergoing intensive induction chemotherapy.

## Materials and methods

### Study design and microbiological definitions

In this single center study, we retrospectively included all patients with AML (excluding acute promyelocytic leukemia, APL) who underwent intensive induction chemotherapy between 2007 and 2015. According to local infection control guidelines MDRO screening was performed for all patients on the day of admission and once weekly thereafter. No additional MDRO screenings were performed in cases of fever or CRP increase. Patients with at least one positive swab for MDRO before or during the hospital stay of induction chemotherapy were defined as colonized. Patients who never had a positive swab for MDRO were defined as noncolonized. Patients who acquired a positive swab after the hospital stay of induction chemotherapy were not further investigated. MDRO were defined as VRE, MRSA and MDRGN. MDRGN had been described previously as Enterobacteriaceae with extended-spectrum β-lactamase (ESBL)-like phenotype and Enterobacteriaceae, Acinetobacter baumannii, and Pseudomonas aeruginosa resistant against piperacillin; any third-generation or fourth-generation cephalosporin ± resistance to fluoroquinolones [7]. MDRGN with resistance against carbapenems have been described as Carbapenem-resistant Enterobacteriaceae (CRE) [8]. The study was performed in accordance with the 2013 Helsinki declaration. Patients provided informed written consent and patient data was provided after approval by the local Ethics Committee (approval number SHN-09-2016). The ethics committee waived the requirement for informed consent for deceased patients. In addition, the majority of patients were also enrolled in the AML registry of the Study Alliance Leukemia (approval number EK 98032010). After ethics approval, patient data was retrieved from the clinical cancer registry of the University Cancer Center (UCT) Frankfurt, complemented by data directly from the medical records and fully anonymized. Data analysis was performed on anonymized data.

### Detection of MDRO and molecular resistance analysis

For MDRO screening culture swabs were transferred from Amies collection and transport medium (Hain Lifescience, Nehren, Germany) onto selective CHROMagar ESBL plates (Mast Diagnostica, Paris, France). Matrix-assisted laser desorption ionization-time of flight analysis (VITEK MS, bioMérieux, Nürtingen, Germany) was used for identification of bacteria isolates, followed by performance of an antibiogram and resistogram using VITEK 2 and/or antibiotic gradient tests (bioMérieux). Carbapenemases detection was performed via PCR analysis and subsequent sequencing from carbapenem-resistant Enterobacteriaceae including the bla genes for carbapenemases NDM, VIM, IMP, OXA-48, OXA-48 like, and KPC as well as OXA-23, OXA-24, OXA-51, and OXA-58 for Acinetobacter baumannii [9, 10]. For VRE detection swabs from Amies collection and transport medium (Hain Lifescience, Nehren, Germany) were transferred onto ChromID VRE agar (bioMérieux).

### Clinical characteristics and definitions

Standard induction chemotherapy was the so-called *7+3*-regime; cytarabine 100mg/m^2^ given intravenous (IV) continuously for 7 days is combined with daunorubicin 60mg/m^2^ given as a 30minute IV infusion on days 3, 4 and 5 [11]. Patients under the age of 60 received a second induction therapy with *7+3* if early blast clearance was achieved in d15 bone marrow blood evaluation or with *HAM protocol* (cytarabine 3000mg/m^2^ was administered by 3-hour IV infusion every 12 hours on day 1 through 3 and mitoxantrone 10mg/m^2^ by 30-minute IV infusion on day 3,4 and 5) if blast clearance was not achieved on d15 bone marrow blood evaluation [12]. Patients above the age of 60 received only a second induction chemotherapy with HAM (with reduced cytarabine dose of 1000mg/m^2^), if the first induction therapy cycle was not sufficient to achieve bone marrow blast clearance on d15 [13]. Blood testing (hematology, liver and kidney function, coagulation, inflammation markers) was performed every other day routinely. All patients received routinely antibiotic prophylaxis with levofloxacin 500mg daily as suggested by current guidelines [5, 14].

Grade 4 neutropenia was defined as a neutrophil count below 500/μL [15]. A day with *f*ever was defined if body temperature was measured ≥38.3°C once or ≥38.0°C on two consecutive days [16]. If fever or a significant increase of C-reactive protein (CRP) (doubling of CRP level and absolute value above 5 mg/dl, norm < 0.5 mg/dl) was diagnosed antibiotic prophylaxis was replaced by intravenous broadband antibiotics. Patients with evidence of MDRO colonization were treated with respect to the resistogram; all others received the β-lactam antibiotic piperacillin/tazobactam empirically. If catheter or soft tissue infection was suspected vancomycin was given additionally or teicoplanin or linezolid if evidence of VRE Van-B or Van-A, respectively.

### Statistical analysis

This study was designed as a retrospective cohort study. Patients were followed till death or last contact. Dates of treatment start and finish with induction chemotherapy were assessed separately. Continuous variables are shown as means ± standard deviation and categorical variables are reported as frequencies and percentages. All continuous variables were tested for normality and were analyzed by using the Student´s *t*-test or the Wilcoxon-Mann-Whitney test accordingly. Chi-squared test was used for binary variables. Death rates and day of neutropenia analysis were analyzed by Kaplan-Meier method and curves were compared by log-rank test. Predictors of survival were determined using an univariate Cox regression hazard model. Death was recorded as event. For assessment of independent predictors of survival, a multivariate Cox regression hazard model with forward stepwise (likelihood ratio) entry was used, factors with p<0.1 in the univariate analysis were included. Statistical analysis was performed with SPSS (Version 22.0, IBM, Armonk, NY).

## Results

### Baseline characteristics and microbiological findings

**Fig 1:**
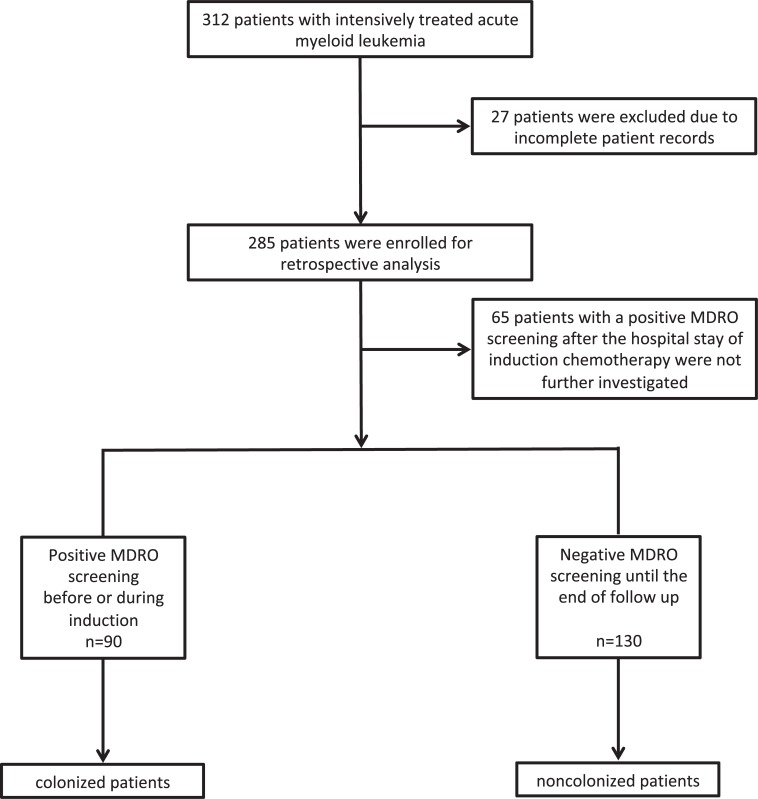
Flow sheet for screening, enrollment and allocation.

312 patients diagnosed with AML between 2007 and 2015 that went on to receive an intensive induction therapy after diagnosis were included in this retrospective analysis **(Fig 1)**. 27 of these patients had to be excluded due to incomplete patient records, 65 patients with a positive MDRO screening after the hospital stay of induction chemotherapy were not further investigated. Patients with a positive MDRO screening before or during the hospital stay of induction chemotherapy were defined as colonized patients (n = 90), patients that never had a positive MDRO-screening were defined as noncolonized patients (n = 130). The two cohorts showed no significant differences in the baseline characteristics **(Table 1)**. Cytogenetic and molecular genetic data were combined into risk groups according to the 2010 ELN guidelines [17]. Favorable, intermediate and adverse genetic groups were well balanced between colonized and noncolonized patients. The microbiological analysis showed that within the colonized patients the most frequently found MDRO was VRE with 67 patients (74.4%) followed by 18 ESBL-positive patients (20%) with or without resistance to fluoroquinolones (ESBL/±FQ). CRE was found in 12 patients (13.3%), MRSA was only found in 2 of our patients (2.2%). 9 patients (10%) were colonized by more than 1 MDRO **(Table 2)**.

**Table 1:** Baseline characteristics.

Characteristic	All patients	Colonized	Noncolonized	*p* Value
Number of patients (n, %)	220 (100)	90 (40.9)	130 (59.1)	** **
Median age (median, range)	60.5 (18–85)	59 (18–85)	61.5 (19–82)	ns
Male sex (n, %)	111 (50.5)	52 (57.8)	59 (45.4)	ns
Favorable genetic group (n, %)	44 (20.3)	15 (17)	29 (22.5)	ns
Intermediate genetic group (n,%)	135 (62.2)	61 (70.5)	74 (57.4)	ns
Adverse genetic group (n,%)	37 (17.0)	11 (12.5)	26 (20.1)	ns
Peripheral blood blast count* (median, range)	28 (0–98)	31.5 (0–93)	26 (0–98)	ns
Bone marrow blast count* (median, range)	60 (5–96)	60 (5–95)	60 (5–96)	ns
Lactate dehydrogenase* (median, range)	406.5 (128–4803)	426 (128–3819)	402 (150–4803)	ns
Thrombocytes* (median, range)	53 (5–590)	49.5 (10–590)	54.5 (5–548)	ns
Leukocytes* (median, range)	14.62 (0.38–340)	14.12 (0.38–340)	15.19 (0.78–311.82)	ns
Hemoglobin* (median, range)	9.1 (3.5–16.2)	9.25 (4.3–16)	8,85 (3.5–16.2)	ns
C-reactive protein* (median, range)	2.94 (0.02–34.03)	3.28 (0.02–34.03)	2.73 (0.02–29.87)	ns

*at time of diagnosis. All p-values reported are two-sided. Statistical significance was defined as p≤0.05.

**Table 2:** Microbiological findings.

Characteristic	Colonized	Noncolonized
Number of patients (n, %)	90 (40.9)	130 (59.1)
VRE (n, %)	67 (74.4)	0
ESBL/±FQ (n, %)	18 (20)	0
CRE (n, %)	12 (13.3)	0
MRSA (n, %)	2 (2.2)	0
≥2 MDRO (n, %)	9 (10)	0

VRE indicates vancomycin-resistant enterococcus, ESBL/±FQ enterobacteriaceae with extended-spectrum b-lactamase phenotype with or without flourquinolone resistance, CRE indicates carbapenem-resistant enterobacteriaceae, MRSA methicillin-resistant staphylococcus aureus and MDRO indicates multidrug-resistant organism.

### Clinical findings

46 (51.1%) of the colonized patients and 61 (46.9%) of the noncolonized patients received a single induction therapy (p = 0.636), the others received two induction chemotherapy cycles respectively (48.9% vs. 53.1% p = 0.636) **(Table 3)**. 45 of the colonized patients (50%) and 61 of the noncolonized patients (46.9%) received an allogenic stem cell transplantation (SCT) as consolidation therapy (p = 0.682). The median time to allogenic SCT was 134 days (range 28–2349) for the colonized and 111 days (range 36–1865) for the noncolonized patients (p = 0.627), the median length of the hospital stay was 50 days (range 15–93) for colonized and 49 days (range 8–82) for noncolonized patients (p = 0,485). Colonized patients had more days with fever than noncolonized patients (6 days [0–28] vs. 5 days [0–31], p = 0.01). 23 (25.6%) of the colonized AML patients required treatment on ICU, which is a significantly increased proportion compared to the noncolonized patient cohort (18 patients, 13.9%, p = 0.035). Colonized and noncolonized patients had equal hemoglobin levels, white blood cell counts and thrombocytes throughout the hospital stay of induction chemotherapy. Colonized patients had significantly higher median CRP levels than noncolonized patients (4.92 [0.2–27.49] vs. 3.58 [0.4–34.32], p = 0.005). There was no difference in days with neutropenia during the hospital stay between the two cohorts, 21 days (range 1–86) for colonized patients and 21,5 days (range 0–52) for noncolonized patients (p = 0.566). To analyze a possible impact of MDRO-colonization on AML specific features, we compared the day 15 blast clearance and cytomorphological complete remission (CR) rates of colonized and noncolonized patients. No significant difference was found in this analysis **(Table 3)**.

**Table 3:** Clinical findings.

Characteristic	Colonized	Noncolonized	*p* Value
Number of patients (n, %)	90 (40.9)	130 (59.1)	
Single induction chemotherapy (n, %)	46 (51.1)	61(46.9)	ns
Double induction chemotherapy (n, %)	44 (48.9)	69 (53.1)	ns
Allogenic stem cell transplantation as consolidation therapy (n, %)	45 (50)	61 (46.9)	ns
Time to allogenic stem cell transplantation (median, range)	134 (28–2349)	111 (36–1865)	ns
Length of hospital stay (median, range)	50 (15–93)	49 (8–84)	ns
Days with fever (median, range)	6 (0–28)	5 (0–31)	0.01
Patients requiring treatment on intensive care unit (n, %)	23 (25.6)	18 (13.9)	0.035
Hemoglobin g/dl* (median, range)	9.25 (4.3–16)	8.85 (3.5–16.2)	ns
Leukocytes/nl* (median, range)	0.67 (0.12–12.5)	0.62 (0.11–73.06)	ns
Thrombozytes/nl* (median, range)	28 (8–143)	28 (7–185)	ns
Day 15 bone marrow blast clearance (n, %)	54 (60)	86 (66.2)	ns
Complete remission after induction chemotherapy (n, %)	59 (65.6)	96 (73.9)	ns
C-reactive protein (median, range)	4,92 (0.2–27.5)	3,58 (0.4–34.3)	0.005
Days with Grade 4 neutropenia (median, range)	21 (1–86)	21,5 (0–52)	ns
Day 15 bone marrow blast clearance (n, %)	54 (60)	86 (66.2)	ns
Complete remission after induction chemotherapy (n, %)	59 (65.6)	96 (73.9)	ns

*Median of values recorded during hospital stay of induction chemotherapy. All p-values reported are two-sided. Statistical significance was defined as p≤0.05.

### Outcome

We hypothesized that colonization with MDRO might be of prognostic value in AML patients. Therefore, the overall survival (OS) rates of colonized and noncolonized AML patients were compared. There was no statistical difference between the two groups (hazard ratio (HR) 1.216, 95% confidence interval (CI) 0.839–1.762, *P* = 0.301). When investigating the subgroups of MDRO (VRE, ESBL/±FQ and CRE) we found that CRE-colonized patients showed a poor survival rate (HR 3.129, 95% confidence interval (CI) 1.596–6.134, *P* = 0.001). Death rate in these patients was significantly higher at 2 months (33.3% versus 8.4% p = 0.007), at 3 months (33.3% versus 10%, p = 0.015), at 1 year (66.6% versus 25.4%, p<0.001) and at 2 years (75% versus 41.5%, p<0.001). The most common cause of death in these patients was sepsis, followed by disease progression.

To further analyze CRE-colonization as a prognostic parameter in AML patients undergoing intensive induction chemotherapy a multivariate Cox regression model with forward stepwise likelihood ratio was performed. The nominal dichotome variables male gender, age above 60 years, adverse-risk AML, day 15 bone marrow blast clearance, allogenic SCT as consolidation therapy and colonization with a MDRO or one of the subspecies were included in this model. As shown in **Table 4** age above 60 years, day 15 bone marrow blast clearance, allogenic SCT as consolidation therapy and colonization with CRE were independently associated with OS.

**Table 4:** Univariate and multivariate analysis associated with survival in AML patients. VRE indicates vancomycin-resistant enterococcus, ESBL/±FQ enterobacteriaceae with extended-spectrum b-lactamase phenotype with or without flourquinolone resistance, CRE indicates carbapenem-resistant enterobacteriaceae, MRSA methicillin-resistant staphylococcus aureus and MDRO indicates multidrug-resistant organism. CI indicates confidence interval and HR hazard ratio. All p-values reported are two-sided. Statistical significance was defined as p≤0.05.

Parameter	HR	95% CI	*P* value	HR	95% CI	*P* value
	Univariate analysis			Multivariate analysis		
Male gender	1.311	0.906–1.895	0.151			
Age > 60	5.530	3.154–9.696	<0.001	4.857	2.144–11.006	<0.001
Adverse genetic group AML	1.608	0.264–0.663	0.043			
Day 15 bone marrow blast clearance	0.419	0.264–0.663	<0.001	0.353	0.196–0.636	<0.001
Stem cell transplantation as consolidation therapy	0.300	0.203–0.445	<0.001	0.531	0.299–0.943	0.031
Colonization with MDRO	1.216	0.839–1.762	0.301			
Colonization with VRE	1.136	0.754–1.711	0.543			
Colonization with ESBL/±FQ	1.439	0.862–2.401	0.164			
Colonization with CRE	3.129	1.596–6.134	0.001	3.137	1.299–7.574	0.011

## Discussion

Several studies have analyzed the clinical impact of MDRO in patients with hematologic malignancies (HM). These studies differ in the MDRO that were analyzed, in the underlying disease of the study population and in the treatment of these patients [6, 18–31]. Most of these studies analyzed the impact of MDRO infections rather than the effect of MDRO colonization only. To our knowledge the present study is the first to investigate the role of MDRO colonization in AML patients receiving standard intensive induction chemotherapy. This is of particular interest given the increasing spread of MDRO colonization worldwide.

We found 90 patients with a positive MDRO screening before or during the hospital stay of induction chemotherapy (colonized patients) and 130 patients that never had a positive MDRO-screening (noncolonized patients). We excluded the 65 AML patients that acquired MDRO colonization after the initial hospital stay of induction chemotherapy because this is potentially a mixed population: they either were MDRO-colonized during the hospital stay (and remained undetected) or they acquired MDRO-colonization as outpatients or during subsequent hospital stays (such as during consolidation chemotherapy). We have therefore excluded these patients from further analysis and concentrated our efforts on the two clearly defined and separated patient cohorts described in the manuscript. Ideally, an age-matched control population with unrelated disease could allow to see if the rates of colonization of patients getting hospitalized for AML induction treatment are comparable. However, as these patients would significantly differ in their antibiotic use, immune status and regular MDRO screening intervals such an analysis would be of limited value and was therefore not performed.

Our results show that colonized patients have a higher demand for treatment on ICU than noncolonized patients. This did not prolong the length of the hospital stay. The higher incidence of fever in MDRO colonized patients has been previously reported by several groups, including higher mortality—due to fatal infections—and/or prolonged length of hospital stay for patients with HM [6, 18, 23]. Our observation underlines the importance of microbiological screening in feverish neutropenic patients not only with blood cultures but also with repeatedly nasal, oral and rectal screenings swabs, since persisting fever can be a sign of MDRO colonization.

CRP is an acute phase protein that is elevated in the blood plasma in response to inflammation. High values predict poor prognosis in cancer patients with febrile neutropenia [32, 33]. In our study we analyzed the CRP levels of colonized and noncolonized patients and found higher CRP levels in colonized patients. These results taken together with the increased days of fever indicate that colonized AML patients under intensive induction chemotherapy might be at high risk for infections and death.

Several studies revealed associations between the intestinal microbiome and chemotherapy sensitivity. Lehouritis *et al*. showed *in vitro* and also *in vivo* evidence that Escherichia coli (E. coli) reduces anti-tumor activity of gemcitabine [34]. Viaud *et al*. showed that the intestinal microbiota modulates the anticancer activity of cyclophosphamid [35]. Thus, we were curious to see whether colonization has an impact on treatment response rates in AML patients. In our study no difference in early response rates in terms of bone marrow blast clearance on day 15 or cytomorphological CR rates after induction chemotherapy were found **(Table 4)**.

The median overall survival did not differ between colonized and noncolonized patients. Of particular interest was the subgroup analysis for VRE-colonized patients. Recent studies in our institution showed a survival disadvantage for VRE-colonized patients with HM that underwent autologous SCT [26]. Interestingly, these patients did not die during neutropenia after SCT but mainly during the first 3 months after discharge from hospital. Other studies analyzed the role of BSI with VRE in patients with HM and showed increased mortality for those patients [24, 25]. In our study no significant difference in OS between VRE-colonized and noncolonized AML patients was found. AML patients are repeatedly re-admitted back to hospital after induction therapy is completed for consolidation treatment. Thus, they are persistently under high surveillance after induction chemotherapy and appropriate antibiotic treatment is usually rapidly started. These circumstances may explain the different impact of VRE on AML mortality.

Subgroup analysis for ESBL/±FQ-colonized patients showed no difference with respect to overall survival when compared to noncolonized patients. Cornejo-Juarez *et al*. showed a significant survival disadvantage for 100 hematological patients with BSI by ESBL E. coli when compared to 100 patients with BSI by cephalosporin-susceptible E. coli [18]. Our study however focuses on MDRO colonization and certainly not every colonization leads to BSI. Altogether our ESBL/±FQ cohort is not large enough to draw safe conclusions about the impact of ESBL/±FQ-colonization on OS.

**Fig 2:**
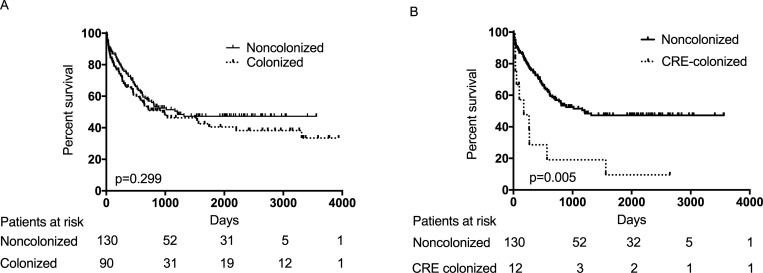
Kaplan-Meier curves for overall survival (OS). **A.** OS of colonized (dotted line) and noncolonized (solid line) patients. **B.** OS of CRE-colonized (dotted line) and noncolonized (solid line) patients.

A small but important subgroup of patients was colonized by CRE (12 patients, 13.3%). For those patients we observed a significant survival disadvantage with respect to 60d-, 90d-, 1y- and 2y OS rates. Death was mostly due to lethal infections (9 out of 12). Trecarichi *et al*. analyzed the clinical impact of BSI with Carbapenem-resistant *Klebsiella pneumonia* (CRKP) in patients with HM in Italy [19]. These 161 patients (of which 119 suffered from AML) showed a high 21-day mortality rate of 52.2%. Micozzi *et al*. analyzed 22 CRKP-positive patients (12 AML patients and 10 with other HM) with different treatment protocols [21]. 10 of them died with evidence of CRKP bacteremia, all of them had AML. Finally Jaiswal *et al*. observed prospectively 225 consecutive patients for CRE-colonization. Patients with HM that underwent treatment were analyzed for a period of 28 months. TRM in AML patients was only seen in CRE-colonized patients [27]. Our study is in accordance with this finding in a complementary approach as we have found that AML patients colonized with CRE have the worst outcome **(Fig 2)**. However, the small number of AML patients colonized with CRE limits the conclusions from our analysis. A multicenter analysis with larger patient numbers is required to confirm our findings.

In conclusion we identified MDRO colonization to significantly determine the clinical course of AML patients undergoing intensive induction chemotherapy. The infectious complication resulting from these MDRO can be managed by initiation of appropriate antibiotic therapy and—if required—ICU treatment. However, in cases of colonization with highly resistant organisms like CRE, our data highlight the importance of appropriate isolation measures and intensive MDRO screenings to avoid MDRO transmission between patients and to detect these high-risk patients early.

## Supporting information

S1 Table Raw data. (XLSX)

## Abbreviations:

AML: Acute myeloid leukemia
BSI: Bloodstream infection
CI: Confidence intervall
CR: Complete remission
CRE: Carbapenem-resistant enterobacteriaceae
CRKP: Carbapenem-resistant Klebsiella pneumonia
CRP: C-reactive protein
ESBL/±FQ: Extended-spectrum b-lactamase producing enterbacteriaceae with or without resistance to fluoroquinolones
E. coli: Escherichia coli
HM: Hematologic malignancies
HR: Hazard ratio
ICU: Intensive care unit
IV: Intravenous
MDRGN: Multidrug-resistant gram-negative bacteria
MDRO: Multidrug-resistant organism
MRSA: Methicillin-resistant staphylococcus aureus
OS: Overall survival
SCT: Stem cell transplantation
TRM: Treatment related mortality
VRE: Vancomycin-resistant enterococcus
